# Hypoglycemia and associated comorbidities among newborns of mothers with diabetes in an academic tertiary care center

**DOI:** 10.3389/fped.2023.1267248

**Published:** 2023-10-13

**Authors:** Maha Bamehrez

**Affiliations:** Pediatric Department, King Abdul Aziz University Hospital, Jeddah, Saudi Arabia

**Keywords:** hypoglycemia, neonatal, sepsis, glucos, newborn, morbididty, large for gestational age, small for gestational age

## Abstract

**Background:**

Hypoglycemia is considered the common metabolic problem in newborns with serious long-term sequelae. This study evaluates the incidence of hypoglycemia in the newborns of mothers with diabetes mellitus and assesses the comorbidities that affect the newborns of mothers with gestational diabetes compared with the newborns of mothers with pregestational diabetes mellitus.

**Methods:**

This retrospective cohort study was conducted between January-2018 and December-2020. All admissions to the hospital nursery of the newborns of diabetic mothers with diabetes mellitus were included.

**Results:**

The study comprised 1,036 mothers with diabetes, of the newborns of mothers with pregestational diabetes, 22% had hypoglycemia, and of mothers with gestational diabetes, 12%. Mothers with pregestational diabetes had a significantly higher risk of needing an emergency cesarean section (OR: 2.1, 95% CI: 1.3–3.4); and of having a baby who is large for its gestational age (OR: 9.5, 95% CI: 2.6–35.5), must be admitted to the NICU (OR: 2.9, 95% CI: 1.5–5.6), has respiratory distress syndrome (OR: 3.3, 95% CI: 1.5–7.4), and needs gavage feeding (OR: 3.5, 95% CI: 1.4–8.9).

**Conclusion:**

About 13% of the newborns of mothers with diabetes had hypoglycemia. Significantly more of these newborns were of mothers with pregestational diabetes than of mothers with gestational diabetes. Newborn of mothers with pregestational diabetes mellitus have the risk of large weight and neurological problems, such as sucking difficulties, length of hospital stay, NICU admission, and respiratory distress syndrome.

## Introduction

Glucose is considered the most important source of energy for neonates. Healthy newborns must keep normal glucose levels for their growth and development. In the uterus, fetus not only utilize glucose immediately but also store some glucose in preparation for birth (1, 2). Thus, healthy newborns can maintain normal blood glucose levels for the first few days after birth until they adapt to feeding properly. An imbalance in the glucose supply or low glucose production may explain low blood glucose levels (1). Other explanations include increased metabolic demand, excessive insulin production, and defects in the pituitary or adrenal regulatory mechanisms.

Hypoglycemia in newborns is a common metabolic disturbance and usually occurs in babies whose mothers have diabetes mellitus or a hormonal imbalance (3). Hypoglycemia in neonates, however, is defined as low blood glucose levels (<47 mg/dl) in the first hours or days after birth (4). Studies have proven that blood glucose below 47 mg/dl for five or more days is directly associated with neurodevelopmental impairment at an early age (5, 6). Moreover, low blood glucose levels in neonates have been found to be directly associated with apnea, irritability, lethargy, seizures, and brain damage. The American Academy of Pediatrics (AAP) has developed useful guidelines for the screening and management of neonatal hypoglycemia. They state that a plasma glucose concentration of more than 25 mg/dl is generally safe in a healthy newborn. In fact, this value is considered adequate for up to 4 h. Beyond this, it must exceed 35 mg/dl in the first 24 h otherwise, an intervention is required (7).

Studies have shown that hypoglycemia also occurs in newborns (3, 7). These newborns include those who are small for their GA due to intrauterine growth restriction, who are large for their actual GA, who are born with or develop sepsis or a syndrome within the first month of life, and whose mothers have diabetes. Fetal hyperinsulinism, a condition of high utilization of glucose, often occurs in newborns of mothers with diabetes (IMD). Postnatally, this high consumption of glucose in the fetal stage can lead to hypoglycemia in the newborns at birth or shortly after (3, 4). The literature has observed that newborns of mothers with diabetes (IMD) have a higher risk of developing neonatal complications than newborns of mothers who did not have or did not develop diabetes during pregnancy. Studies have proven that chronic, or pre-gestational diabetes mellitus (PGDM), is significantly associated with unfavorable fetal and neonatal comorbidities such as pulmonary disease, neurological complications, congenital anomalies, and feeding difficulties (3, 5, 6, 8). Hence, neonates born to mothers with diabetes mellitus (DM) should have postnatal glucose monitoring and management until metabolic stability is assured.

The aim of the current study, therefore, was to assess the incidence of hypoglycemia in newborns of mothers with diabetes born in King Abdulaziz University Hospital in Jeddah. The further aim was to evaluate which comorbidities are associated with newborns of mothers with pre-gestational diabetes mellitus (PGDM) and mothers with gestational diabetes (GD).

## Materials and methods

This retrospective study of a cohort of patients at King Abdulaziz University Hospital (KAUH), Jeddah, Saudi Arabia, followed the guidelines of the Declaration of Helsinki. The research ethics committee at the Unit of Biomedical Ethics, King Abdulaziz University, Faculty of Medicine, Jeddah, Saudi Arabia, approved this study (approval number: HA-02-J-008).

### Participants

The current study included all nursery admissions for diabetic mothers, whether gestational or pregestational diabetes mellitus, from January 2018 to December 2020. Out born newborns, preterm newborns whose Gestational age (GA) was less than 34 weeks, and newborns of unbooked mothers were excluded. Gestational diabetes mellitus (GDM) is defined as a high blood sugar level that is first recognized during pregnancy while PGDM, diagnosed before pregnancy, can be type I or type II diabetes mellitus, which occurs due to metabolic, hereditary, or environmental causes (9).

### Study design

This study retrieved the electronic medical records of all eligible patients in order to collect the maternal and infant characteristics required for the study. Any information missing from the electronic records or the paper medical files was requested from the medical filing system. At KAUH, the annual number of births ranges from 2,500 to 4,000 deliveries, and policy recommends a hospital stay of 24 h for spontaneous vaginal delivery.

### Newborn care at KAUH

KAUH policy recommends that babies be roomed-in with their mothers soon after birth, not only to encourage breastfeeding and help build the confidence of the mothers in caring for their babies but also to maintain the homeostasis of the newborn. Babies should not be separated from their mothers for more than 2 h for medical reasons. For example, when the infant must be kept in the Neonatal intensive care unit (NICU) or the nursery room for other reasons (e.g., phototherapy), the mother should be asked to come to the nursery for breastfeeding.

### Maternal characteristics

The following data were collected: age; nationality; booking status; and GA (GA), which was calculated based on the last menstrual period, a prenatal ultrasound, and/or pregnancy testing. Type of diabetes (pre-gestational or gestational), the method of diabetic control (diet or drug), and the presence of medical comorbidities, such as hypertension and thyroid disease, were also recorded. Gravida (number of pregnancies that a woman had regardless of the duration, including the present pregnancy) and the method of delivery [standard vaginal delivery (SVD), elective cesarean delivery (LCD), or emergency cesarean delivery (ECD)] was recorded.

### Newborn characteristics

Newborn characteristics, including gender, birth weight (in grams), and Apgar scores, were assessed at 5 min after delivery. newborns weighing more than the 90th percentile for GA were classified as large for GA (LGA). Intra-uterine growth restriction (IUGR) or small for GA (SGA) was determined when birth weight was below the 10th percentile for GA.

All information on the need for resuscitation and reasons for admission to the NICU during the first 72 h of life was retrieved. Information about the development of early onset of sepsis (first 72 h of age) and hyperbilirubinemia was collected. The need for gavage feeding (unable to take oral feeding due to weak sucking reflex) was also recorded. Furthermore, data regarding congenital anomalies and their type were also registered. The diagnosis for cardiac anomalies was confirmed by echocardiogram, whereas a central nervous system (CNS) anomalies diagnosis was confirmed by brain image (US, MRI, CT SCAN). In addition, the diagnosis of renal anomalies was confirmed by ultrasound (US). The clinical diagnosis of respiratory distress that required respiratory support was documented. However, the presence of shoulder dystocia (also called birth injury; it occurs when the fetal shoulder is impacted against the pelvic bone during delivery) was recorded. The length of the infants’ hospital stays (in days) and the baby's outcome (either discharged or dead) were also collected.

### Newborn glucose level

In our study, a blood glucose level of less than 46 mg/dl during the first 36 h of life was considered hypoglycemia. KAUH policy does not recommend routine blood sugar screening for term-healthy newborn babies. Policy does recommend, however, that several infant characteristics be monitored to identify newborns at risk of developing hypoglycemia. These are a mother with diabetes, preterm birth (GA: less than 37 weeks), LGA, SGA, IUGR, signs of infection, resuscitation given at birth, and inadequate feeding. The same policy recommends monitoring for signs of hypoglycemia in newborns who, for example, experience hypothermia, have tremors, are lethargic, or have tachypnea.

### Nutrition and monitoring protocol

Newborns at risk of hypoglycemia should be breastfed or bottle-fed with breast or formula milk within the first hour of birth. The KAUH algorithm requires measuring the blood glucose levels of newborns at risk at 2 h of age and then every 3 h (or before feeding) until regular feeding is established and the blood glucose level exceeds 46 mg/dl. However, at 2 h of age, newborns with blood sugar levels below 32–36 mg/dl were immediately considered for NICU admission.

### Statistical analysis

Data were collected from the medical records of 1,037 patients. KAUH uses Phoenix (internal hospital software) for recording patient data. This study defined maternal age as the mean of total age and converted GA to an ordinal variable and birth weight to grams. Maternal and infant characteristics were compared between neonates born to mothers with GDM and neonates born to mothers with PGDM. According to the relationship between intrauterine growth and GA, newborns were classified into three groups: LGA; appropriate for GA (AGA); and SGA. Frequencies (percentage), and means with standard deviations were calculated using the chi-square test to compare nominal and categorical variables between two groups, whereas the t-test was used to compare mean values between two groups. Analyses were conducted using PSPP software (GNU PSPP, version 1.6.2) with a 2-sided significance level of *p* < 0.05, *p* < 0.001.

Multiple regression analysis was used to adjust for potential confounding factors (maternal age, nationality, gravida, diabetic treatment, underlying comorbidities, and fetal sex); statistical significance was determined using the 95% CI with statistical significance set at *p* < 0.05. Analyses were performed using STATA software (StataCorp. 2011. Stata Statistical Software: Release 12. College Station, TX, USA: StataCorp LP).

## Results

**Figure 1:**
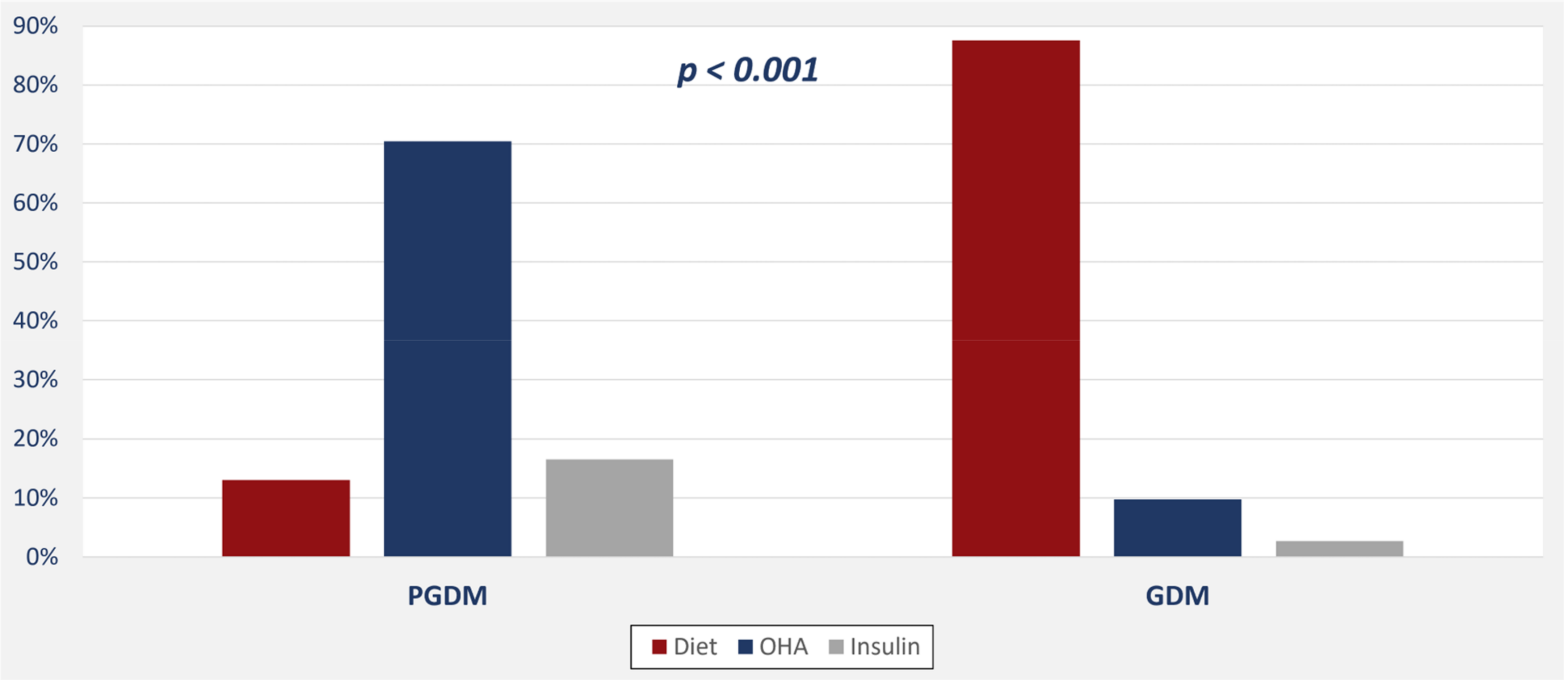
Mode of treatment among mothers with gestational and pregestational diabetes mellitus.

**Figure 2:**
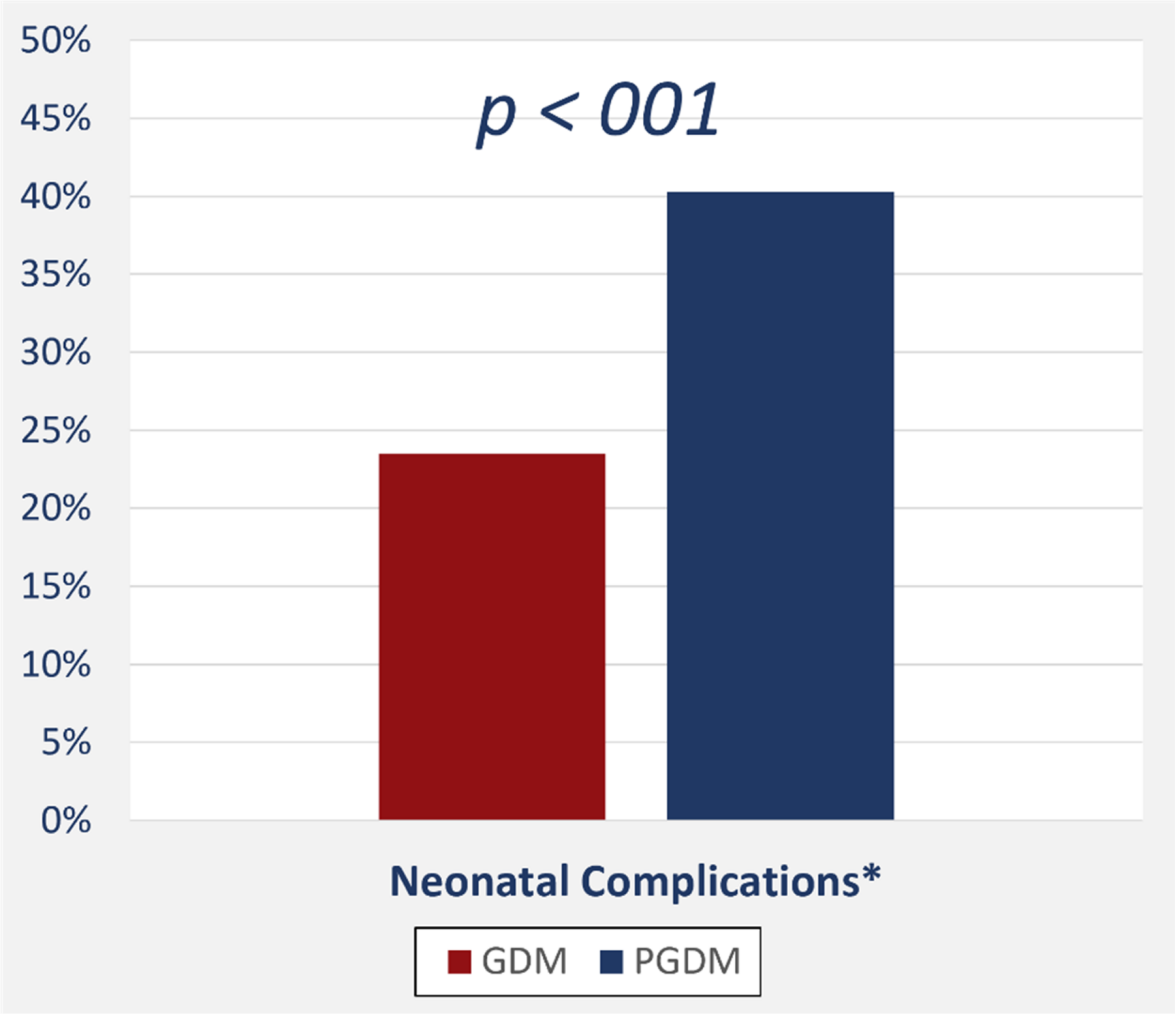
Neonatal complications among newborns of mothers with gestational and pregestational diabetes mellitus.

In all, we evaluated 1,037 pregnant women; 1,036 of these were eligible for the study and classified into one of two groups: GDM or PGDM diabetes. Tables 1, 2 and Figures 1, 2 present maternal and neonatal characteristics. One maternal characteristic, spontaneous labor, was significantly higher (53% higher) in mothers with GDM than the mothers with PGDM. Infant characteristics showed significant between-group differences in sex, Apgar score at 5 min (>9), length of stay, and presence of cardiac anomalies.

**Table 1:** Maternal characteristics according to the type of diabetes mellitus of the mother using the chi-square test for nominal and categorical variables and the *t*-test to compare mean values.

	Mother with GDM (*n* = 912)	Mother with PGDM (*n* = 124)	*p*-value
Maternal age M ± SD	33.10 ± 5.62	33.21 ± 6.11	0.77
Nationality			
Saudi	748 (82%)	83 (67%)	0.08
Non-Saudi	146 (16%)	41 (33%)	0.06
Gravida			
Multigravida	779 (85%)	109 (88%)	0.14
Primigravida	132 (14%)	15 (12%)	0.55
Delivery			
SVD	482 (53%)	35 (28%)	**<** **.** **05**
Cesarean	429 (47%)	89 (50%)	
Emergency	225 (25%)	56 (45%)	0.081
Elective	202 (22%)	33 (27%)	

GDM, gestational diabetes mellitus; PGDM, pre-gestational diabetes mellitus; OHA, oral hypoglycemic agent; SVD, spontaneous vaginal delivery. Bold values are considered significant.

**Table 2:** Neonatal characteristics based on the type of diabetes mellitus of the mother using the chi-square test for nominal and categorical variables and the *t*-test to compare mean values.

	Infant of mother with GDM (*n* = 912)	Infant of mother with PGDM (*n* = 124)	*p*-value
Sex, *n* (%)			
Male	469 (51%)	74 (60%)	**<** **.** **05**
Female	439 (48%)	49 (40%)	**<** **.** **05**
**Birth weight in grams, (M ± SD)**	3,114.15 ± 490.28	3,291.21 ± 688.26	0.07
LGA	55 (6%)	3 (2%)	0.32
SGA	35 (4%)	19 (15%)	0.51
AGA	822 (90%)	102 (82%)	**<** **.** **05**
Apgar score at 5 min, *n* (%)			
>9	691 (76%)	71 (57%)	**<** **.** **05**
7–9	215 (24%)	53 (43%)	0.08
<7	4	4 (3%)	
GA* (M ± SD)	38.68 ± 2.30	37.79 ± 1.80	0.52
Anomalies, *n* (%)			
Cardiac anomalies	31 (3%)	14 (11%)	**<** **.** **05**
CNS anomalies	11 (1%)	1 (1%)	0.07
Renal anomalies	11 (1%)	1 (1%)	0.07
Gavage feeding, *n* (%)	24 (3%)	16 (13%)	0.55
Length of stay, *n* (%)			
One day	510 (56%)	39 (31%)	**<** **.** **05**
Two days	221 (24%)	25 (20%)	
Three days	101 (11%)	28 (23%)	

GDM, gestational diabetes mellitus; PGDM, pre-gestational diabetes mellitus; GA, gestational age; LGA, large for gestational age; SGA, small for gestational age; AGA, appreoperate for gestational age; CNS, central nervous system. Significant *p* value <0.05. (−), The sample size was insufficient to test a significant difference. Bold values are considered significant *p* value <0.05.

### Newborn hypoglycemia

Prevalence of hypoglycemia in the study cohort was 13%. Hypoglycemia prevalence in newborns of mothers with chronic diabetes was 22%, and with gestational diabetes, 12%.

### Fetal and neonatal outcomes

Table 3 shows that respiratory distress syndrome and NICU admission were significantly higher in newborns of mothers with chronic diabetes.

**Table 3:** Neonatal characteristics based on the type of diabetes mellitus of the mother using the chi-square test for nominal and categorical variables and the *t*-test for comparing mean values.

*n* (%)	Infant of mother with GDM (*n* = 912)	Infant of mother with PGDM (*n* = 124)	*p*-value
Sepsis			
Yes	4	2 (2%)	0.32
No	907 (99%)	122 (98%)	
Need for resuscitation			
Yes	0	2 (2%)	0.91
No	912 (100%)	122 (98%)	
Shoulder dystocia			
Yes	0	3 (2%)	0.62
No	912 (100%)	121 (98%)	
Respiratory distress			
Yes	36 (4%)	19 (15%)	**<** **.** **05**
No	876 (96%)	105 (85%)	
NICU admission			
Yes	62 (7%)	27 (22%)	**<** **.** **05**
No	850 (93%)	97 (78%)	
Hyperbilirubinemia			
Yes	197 (22%)	46 (37%)	0.45
No	715 (78%)	78 (63%)	

GDM, gestational diabetes mellitus; PGDM, pre-gestational diabetes mellitus. Significant *p* value <0.05. (−), The sample size was insufficient to test for a significant difference. Bold values are considered significant *p* value <0.05.

### Associations between type of diabetes and fetal and neonatal outcomes

**Figure 3:**
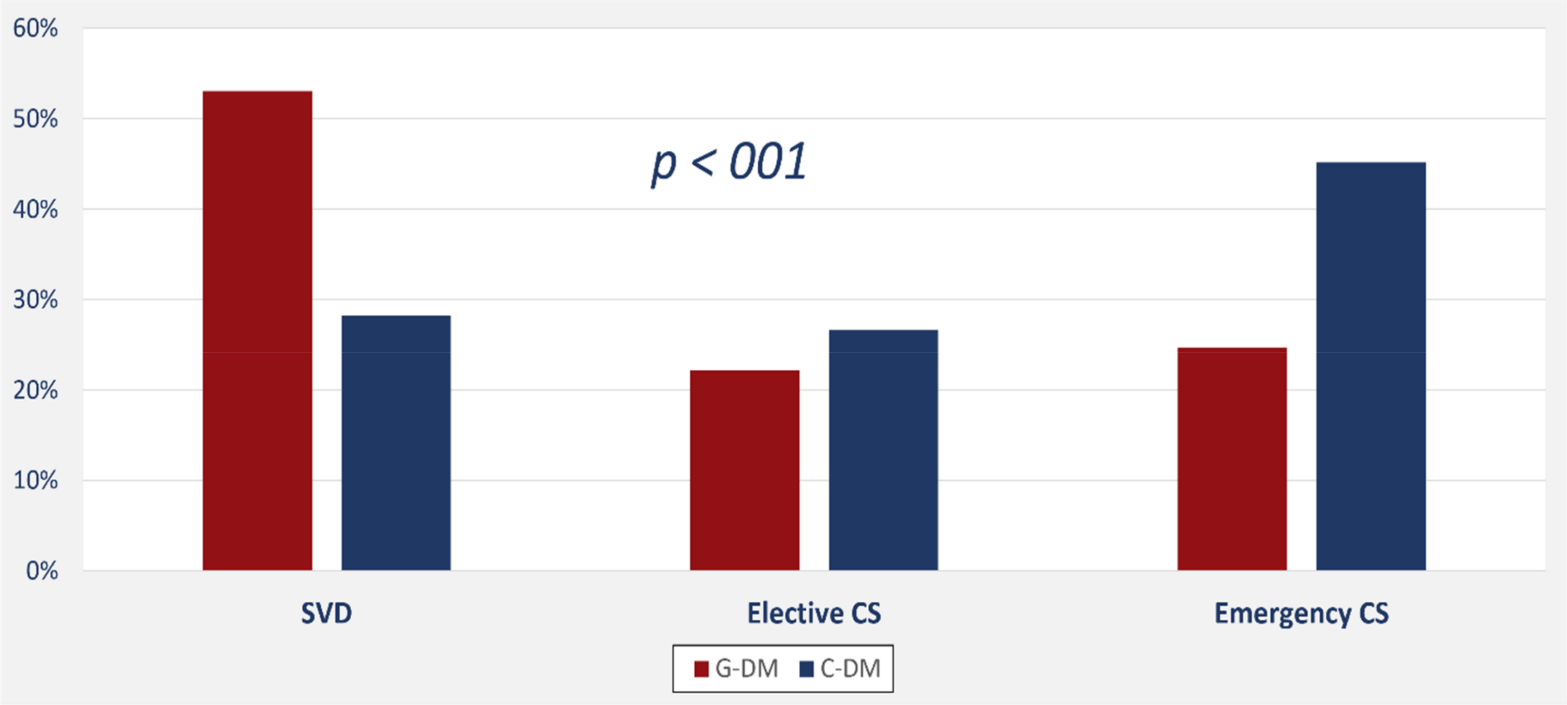
Mode of delivery among mothers with gestational and pregestational diabetes.

PGD is significantly associated with an increased risk of emergency cesarean section (OR: 2.1, 95% CI: 1.3–3.4), large for GA (OR: 9.5, 95% CI: 2.6–35.5), NICU admission (OR: 2.9, 95% CI: 1.5–5.6), respiratory distress syndrome (OR: 3.3, 95% CI: 1.5–7.4), and the need for gavage feeding (OR: 3.5, 95% CI: 1.4–8.9). On the other hand, PGD was not significantly associated with hypoglycemia (adjusted OR: 1.54, 95% CI: 0.84–2.83) or hyperbilirubinemia (adjusted OR: 1.37, 95% CI: 0.83–2.3). Type of diabetes had a significant impact on the mode of delivery with more frequent emergency caesareans among women with PGD (Figure 3). Also, the average length of hospital stay was significantly longer among newborns of mothers with PGD (4.5 days, 95% CI: 3.3–5.8) than newborns of mothers with GD (2.4 days, 95% CI: 1.9–2.8; *p*-value < 0.001).

## Discussion

Neonatal hypoglycemia occurs in newborns of mothers with diabetes due to an impaired gluconeogenesis process. Thus, these newborns have a high risk of developing hypoglycemia (10). The main aim of the present study was to assess the incidence hypoglycemia in newborns of diabetic mothers. The further aim was to evaluate if there are associations between type of maternal diabetes and fetal outcome. Our study found that about 13% of the newborns born at KAUH and whose mothers had diabetes suffered from hypoglycemia. Similarly, Karcaaltincaba et al. 2009 reported a frequency of 14% for newborns among pregnant women with diabetes (11), while other studies found higher frequencies of between 25% and 50% (12, 13). A comparison between the two types of maternal diabetes revealed that 12% of the newborns of mothers with GDM had been diagnosed with hypoglycemia compared with 21% of the newborns of mothers with PGDM. Similarly, other studies found that hypoglycemia is remarkably higher in newborns of mothers with chronic or pregestational diabetes compared with gestational diabetes (14, 15). The Pedersen hypothesis states that maternal hyperglycemia leads to fetal hyperinsulinemia due to overstimulation of the fetal pancreatic cells to produce insulin. In turn, neonatal hyperinsulinemia leads to hypoglycemia, which occurs more frequently in newborns of mothers with a longer duration of diabetes, as in PGDM, when compared with mothers who developed diabetes during pregnancy, as in GDM. This hypothesis may explain our findings (10).

The present study also found that newborns of mothers affected with PGDM are born large of gestational age. This finding is similar to Agarwal 2000 who found that newborns of mothers with a longer duration of diabetes had significantly larger birth weight compared with gestational diabestes mellitus (8). This finding is in line with the notion that the blood glucose crosses the placenta which causes hyperglycemia in the fetus. In turn, this hyperglycemia induces hyperinsulinemia (high insulin production) in the fetus, subsequently increasing the birth weight of the infant (16). Moreover, hyperinsulinemia increases oxidative stress, which causes chronic inflammation and hypoxia in the brain (17). Consequently, this hypoxia would negatively affect brain development and lead to neurobehavioral disorders, as the current study found: maternal PGDM was significantly associated with the infant's need for gavage feeding (18). As a result of neurodevelopmental deficiencies in infant of mothers with PGDM is the immaturity of sucking abilities. Sucking activity requires normal neuromotor function; thus, many studies have reported poor sucking patterns in newborns of mothers with DM due to some degree of neurobehavioral immaturity (3, 19). Hence, many studies found an association between the presence of serum leptin in newborns of mothers with diabetes and feeding difficulties (20). Leptin, the anorexigenic neurotransmitter, possibly counteracts the effect of neuropeptide Y (21). This mechanism may disturb the feeding behaviors of newborns of diabetic mothers. In addition, this abnormality in the sucking process might reflect the actual infant age, which is consistent with a study that found poor sucking activity in premature infants (22). Remarkably, our study found that the average length of infant hospital stay was significantly longer among newborns of mothers with PGDM than of mothers with GDM. This finding parallels another study that suggested the main cause for a prolonged hospital stay was feeding difficulties (23).

Various studies have concluded that glucose imbalances in neonates have several comorbidities, such as, high infant weight, NICU admission, RDS, and neurological and perinatal complications (24–26). Similarly, our study has shown a significant association between newborns of mothers with PGDM and NICU admission, while other studies have concluded that prolonged hypoglycemia could affect the neurodevelopment of the infant and leads to intensive care admission (27, 28). A justification for this relationship is that the longer duration of maternal diabetes, in pre-gestational compared with gestational diabetes, exerts prolonged negative effects on organ development in the offspring, such as in fetal neurodevelopment.

The current study also showed that newborns of mothers with PGDM are at high risk of developing RDS. This finding is not surprising since many studies have proven that the effect of maternal diabetes on the neonate is a susceptibility to many complications, including RDS (28, 29). Furthermore, the present study revealed significant differences between newborns of mothers with PGDM and newborns of mothers with GDM, documenting a higher frequency of cardiac problems in newborns of mothers with PGDM. Likewise, the American College of Obstetricians and Gynecologists (ACOG) reported that ventricular septal defects and transposition of the great vessels are five times higher in newborns of mothers with PGDM (30).

Interestingly, many studies have shown that there is an increased risk of delivery complications and birth injuries in mothers with PGD, which could lead to the need for an emergency delivery, like a cesarean section (31). Similarly, our study reported significant associations between the need for an emergency cesarean delivery and pregestational diabetes in mothers. This finding might explain the significant associations our study recorded between length of hospital stay and mother with PGDM. Likewise, one study from Saudi Arabia found elective CS delivery to be significantly more frequent in mothers with PGDM than in mothers with GDM (32).

This study has several strengths. First, methodologically, this retrospective cohort model provides a large and representative sample with the same data-gathering methods. Second, the data were extracted from digital- and paper-based records, which have been proven to be reliable. Third, our study used multiple regression analysis to compare associations between multiple groups, while adjusting for various potential confounders. This study also has some limitations, such as, its retrospective nature; some details concerning the diagnosis of PGDM, such as HA1c, are lacking, which possibly could have described more associations between the variables. The sample size could also be considered a limitation; however, we included all eligible pregnant women with DM admitted to KAUH during the study period. The current data is limited since it represents a single center. It may not be possible to generalize our results.

In conclusion, about 13% of newborns of mothers with diabetes suffer from hypoglycemia. Of these, 12% of newborns of mothers with GDM were diagnosed with hypoglycemia, and with PGDM, 21%. However, newborns of mothers with PGDM have a greater risk of high birth weight; neurological problems, such as sucking difficulties; prolonged hospital stay; NICU admission; and RDS.

## Data Availability

The original contributions presented in the study are included in the article/Supplementary Material, further inquiries can be directed to the corresponding author.
